# Impact of Blood-Flow-Restricted Training on Arterial Functions and Angiogenesis—A Systematic Review with Meta-Analysis

**DOI:** 10.3390/biomedicines11061601

**Published:** 2023-05-31

**Authors:** Mikołaj Maga, Agnieszka Wachsmann-Maga, Krzysztof Batko, Aleksandra Włodarczyk, Paulina Kłapacz, Jakub Krężel, Natalia Szopa, Agnieszka Sliwka

**Affiliations:** 1 Department of Rehabilitation in Internal Diseases, Faculty of Health Sciences, Jagiellonian University Medical College, 31-008 Krakow, Poland; mikolaj.maga@gmail.com; 2 Clinical Department of Angiology, University Hospital in Krakow, 30-688 Krakow, Poland; agnieszka.wachsmann@gmail.com (A.W.-M.); paulina24k@gmail.com (P.K.); jakub.krezel@gmail.com (J.K.); 3 Department of Angiology, Faculty of Medicine, Jagiellonian University Medical College, 31-008 Krakow, Poland; wlodarczyk.aleksandra1212@gmail.com (A.W.); natalia.szopa24@gmail.com (N.S.); 4 Department of Research and Design, Medicine Economy Law Society (MELS) Foundation, 30-040 Krakow, Poland; batko.krzysztof@gmail.com

**Keywords:** blood-flow-restricted training, vascular functions, angiogenesis, endothelium

## Abstract

Despite growing evidence of the significant influence of blood-flow-restricted (BFR) training on different body functions, its impact on the vascular system, especially the arteries, is controversial. Therefore, the objective of our study was to analyze how BFR exercise, compared to other types of exercise without the restriction of blood flow, influences arterial functions and angiogenesis in adults. Studies comparing the effect of BFR versus non-BFR training on arterial parameters were divided into three categories: endothelial function, angiogenesis, and other vasculature functions. The search was based on Cochrane Library, PubMed^®^, and Embase, and 38 studies were included. The meta-analysis revealed a more significant improvement in flow-mediated dilatation (FMD) (*p* = 0.002) and the production of the primary angiogenesis biomarker vascular endothelial growth factor (VEGF) (*p* = 0.009) after BFR compared to non-BFR training (*p* = 0.002). The analysis of the pulse wave velocity, ankle-brachial index, systolic blood pressure, and heart rate did not show significant differences in changes between BFR and non-BFR training. The other parameters examined did not have sufficient data to be included in the meta-analysis. The results obtained present trends that suggest significant impacts of BFR training on endothelial functions and angiogenesis. There is still a lack of multicenter randomized clinical trials including many participants, and such studies are necessary to confirm the advantage of BFR over non-BFR activity.

## 1. Introduction

In the last decade, blood-flow-restricted (BFR) training has increased in popularity, not only among gym centers but also in the medical field [1]. It is based on the combination of impaired blood flow and various exercises or sports, such as walking, jogging, cycling, resistance exercises, or even yoga practice [2,3,4,5,6]. Originating from the Kaatsu technique, it is constantly modified using multiple tools, such as elastic bands, pressure cuffs, tourniquets, and dedicated instruments such as the Kaatsu Master or Vasper devices, to induce blood flow restriction [7,8]. This approach allows for the achievement of muscular adaptations with lower training loads, typically ranging from 20% to 30% of the one-repetition maximum (1-RM), and a higher number of repetitions per set, typically between 15 and 30 repetitions [9]. By utilizing BFR training, sports medicine professionals have an effective means of attenuating weakness and atrophy following musculoskeletal injury or surgical intervention while avoiding excessive stress on healing tissues [10]. This strategy holds significant potential for promoting optimal recovery and rehabilitation outcomes for individuals in these populations. Furthermore, during BFR training, muscle mass and strength increase faster than with regular exercises [11]. It is also often more suitable for people who are unable to perform full-intensity sports activities, due to movement limitations, as BFR training seems to be more efficient and effective [12].

Physical activity is one of the most crucial factors influencing the human vascular system. It leads to a lower resting heart rate and blood pressure as well as an increase in blood oxygenation, improved endothelial functions, and stimulation of the production of proangiogenic factors, improving peripheral vascular flow [13,14]. These phenomena are widely used in the rehabilitation of patients after myocardial infarction or ischemic stroke [15]. Furthermore, walking training is one of the elements of noninvasive treatment in patients with intermittent claudication, accompanied by lifestyle changes and pharmacotherapy [16].

Despite growing evidence of the significant influence of BFR training on different body functions, its impact on the vascular system, especially the arteries, is a matter of controversy [17].

Therefore, the aim of our study was to analyze the results of studies published to date and to answer scientific questions. We aimed to determine whether and how BFR exercise, compared to other types of exercise without blood flow restriction or other active interventions, influences the vascular endothelium in adults, and how BFR exercise influences angiogenesis in adults compared to different types of exercise without blood flow restriction or other active interventions.

## 2. Materials and Methods

### 2.1. Search Strategy

The protocol for this study was registered in PROSPERO (CRD42020222257). It was carried out and reported according to the PRISMA Statement and Cochrane Guidelines [18,19]. We searched three major online databases: Cochrane Database of Systematic Reviews, PubMed, and Embase. Experimental studies published from January 2000 up to November 2022 that met the inclusion criteria were searched, identified, and included in our analysis. The Cochrane database was searched for similar systematic reviews and for reviews, and their reference lists were checked. We used MeSH terms and Emtree terms related to restricted-blood-flow training in PubMed and Embase, respectively. The detailed search strategy is presented in Appendix A. If MeSH Terms or Emtree were not available, the “all fields search” option was used.

### 2.2. Inclusion Criteria

We included studies that were original articles published in peer-reviewed journals in full text; (b) randomized or nonrandomized controlled trials or crossover-designed studies with a study group (at least one) who performed BFR activity, including Kaatsu training, and another group who performed another active intervention/treatment (other forms of BFR activity as a comparator were also accepted); (c) studies conducted in adult humans; (d) studies that presented results related to arterial functions (at least one parameter) such as endothelial functions (flow-mediated dilatation, FMD; intima-media thickness, IMT; reactive hyperemia index, RHI; vascular stiffness, AI/SI/RI, NO), angiogenesis (VEGF, CD31/PECAM-1, CD106/VCAM-1, Von Willebrand Factor), and other vascular functions (Tcpo2, ABI, TBI, CAVI).

### 2.3. Screening

All matching references were imported into the bibliographic software Mendeley v.1.19.8 [20]. The duplicates were removed. During screening, two reviewers independently searched through the titles and abstracts to choose the eligible records. The third reviewer resolved disagreements. An analysis based on full text was performed for all articles that met the inclusion criteria or were of uncertain significance. Two reviewers performed the full text analysis, and an independent reviewer made the final decisions in case of disagreements.

### 2.4. Data Extraction

A data extraction form with categories of information was used to collect data. It included an assessment of the quality and completeness of the data contained in the included studies (Supplementary Table S1). Among the other categories were the study design; clinical population characteristics; type of exercise; exercise protocol, including the duration and load; the type and grade of blood flow restriction; and the types of outcomes and their values. It was performed by two independent reviewers, while the third reviewer solved any differences in the extracted data. At this point, review articles on the examined topic were also searched for relevant references that could have been omitted during screening.

### 2.5. Bias Risk Assessment

The quality of the included studies was assessed based on the Joanna Briggs Institute critical appraisal tools for randomized and nonrandomized prospective studies [21]. The risk of bias was assessed by independent reviewers. In the event of disagreements, there was a discussion and consensus with additional reviewers.

### 2.6. Statistical Analysis

Analyses were performed in R 4.2.2 (R Core Team, Statistical Foundation, Vienna, Austria) using the Metafor and ESC packages. Studies were included if sufficient data were available to calculate the standardized mean change with raw score standardization (SMCR) [22,23]. Separate meta-analyses were conducted for each predefined outcome measure reflecting the acute hemodynamic response. The SMCR was chosen as an effect size measure (Yi) due to a pretest and post-test control group design that was utilized across studies. Yi was calculated as the difference between the SMCR of the treatment and control samples, while the sampling variance was added due to group independence [24]. A conservative estimate for the correlation between measurements was set at 0.7. We used a random-effects modeling approach with a restricted maximum likelihood (REML) estimator [25]. A comparison was illustrated with a forest plot based on the standardized mean change with a 95% confidence interval for each outcome. The heterogeneity across studies was evaluated using the I-squared value and Q test. The *p*-value was considered statistically significant at <0.05 [26].

## 3. Results

### 3.1. Study Selection

**Figure 1:**
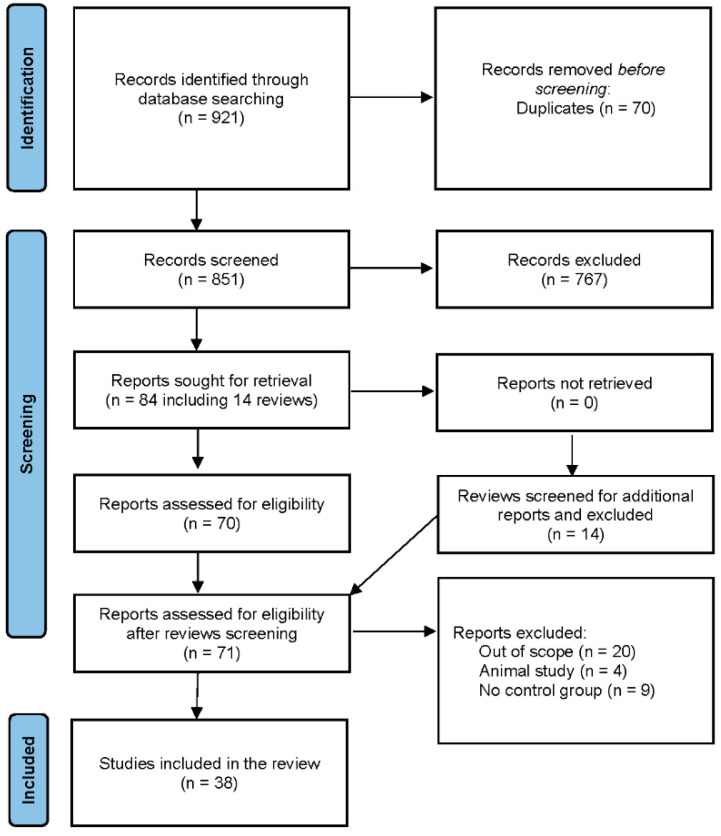
PRISMA flow diagram of the study.

The database search identified 921 records, including 70 duplicates. A total of 851 articles were selected, and 84 of them (including 14 review articles) were sought for retrieval. After searching through the reference list of review articles, 1 missing original article was found and 71 articles were evaluated for eligibility. Thirty-eight of them were included in the final analysis (Figure 1).

### 3.2. Included Studies’ Characteristics

The studies were carried out in 12 countries, mainly in the USA (*n* = 13) and Brazil (*n* = 8) from 2005 [27] until 2022 [28]. Most studies used a crossover (*n* = 21) or RCT (*n* = 13) design. One prospective non-RCT study and three crossover-like studies that were performed on different extremities but within the same participants were included.

There were significant differences in the types of activities: resistance exercise (*n* = 21), treadmill (*n* = 2), walking (*n* = 2), cycling on an ergometer (*n* = 4), cross-training interval exercise (*n* = 1), handgrip (*n* = 5), squats and push-ups (*n* = 1), and yoga (*n* = 1). Additionally, the pressure used for blood flow restriction differed between the included studies, ranging from 45 mmHg to 220 mmHg, and most cases used values equal to or above the participant’s systolic blood pressure, causing temporary ischemia (*n* = 24). The number of sessions differed, but in 21 studies, the results were obtained based on only one session.

The total number of subjects in all studies included in this systematic review was 658 participants (72% male) with a mean age of 39.03 (±3.53) years. In most of the studies, the participants did not present any comorbidities, except for two studies conducted in female participants with hypertension [29,30] and one study conducted in patients with coronary arterial disease [31] (Supplementary Table S1).

### 3.3. Endothelial Functions

#### 3.3.1. Flow-Mediated Dilatation

Fourteen studies examining FMD changes were identified, but six of them were not eligible for statistical analysis, due to insufficient data [5,28,32,33,34,35]. In studies excluded from the meta-analysis, Maga et al. [28] found that FMD increased after BFR activity, while two studies showed a decrease [32,34], and the rest did not detect any significant changes.

**Figure 2:**
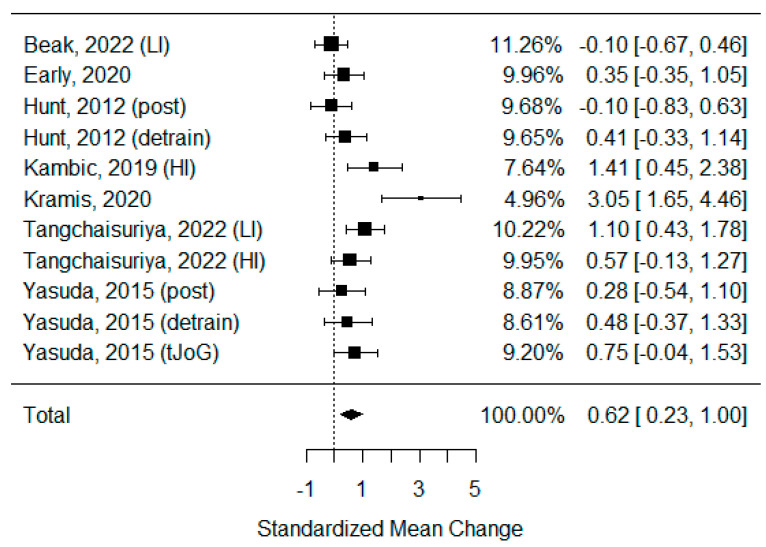
Forest plot describing the effects of BFR exercise on FMD values (post—measured immediately after exercise, detrain—measured up to 1 h after exercise, LI—low-intensity BFR exercise, HI—high-intensity BFR exercise). Beak 2022 [3], Early 2020 [36], Hunt 2012 [37], Kambic 2019 [31], Kramis 2020 [38], Tangchaisuriya 2022 [39], Yasuda 2015 (post/detrain) [40], Yasuda 2015 (tJoG) [41].

As some of the studies consisted of different subgroups performing different types of exercise or/and were assessed at multiple time points, all variants were pulled, resulting in the use of 11 trials to examine the differences between the effects of BFR and non-BFR activity on flow-mediated dilation. The model estimate was 0.617 (CI 0.235, 1.000, *p*-value = 0.002). The heterogeneity was high (I-squared: 62.72%, *p*-value = 0.003) [Figure 2].

#### 3.3.2. Reactive Hyperemia Index

Only three studies analyzed the impact of BFR exercise on the RHI [28,39,42], but the data provided were insufficient for calculation. None of the studies reported any significant changes in RHI due to BFR exercise.

#### 3.3.3. Vascular Stiffness Parameters

Studies analyzing six parameters of vascular stiffness were identified: the augmentation index (AI), corrected AI (AI75), Systemic Vascular Resistance (SVR), Pulse Wave Analysis (PWV), Large-Artery Elasticity Index (LAEI), and Small-Artery Elasticity Index (SAEI). In the case of AI, AI75, SVR, LAEI, and SAEI data were not sufficient to perform calculations:

- AI: Four studies were identified [43,44,45,46], but only Amorim et al. showed a more decisive influence of BFR over non-BFR exercise [43].
- AI75: None of the three identified studies reported any significance in the change in AI75 between BFR and non-BFR exercise [28,43,45].
- SVR: According to Karabulut et al., BFR exercise is more effective for decreasing SVR than low-intensity non-BFR exercise but less effective compared to high-intensity non-BFR training [47]. The rest of the studies presented no significant differences regardless of the exercise type [30,48].
- LAEI: None of the studies analyzing changes in large artery stiffness presented any significant changes [47,48].
- SAEI: Small-artery elasticity improved significantly more after BFR compared to high-intensity non-BFR but not low-intensity non-BFR exercise [48]. Additionally, for push-up and squat exercises, BFR training was more effective for increasing SAEI [47].

**Figure 3:**
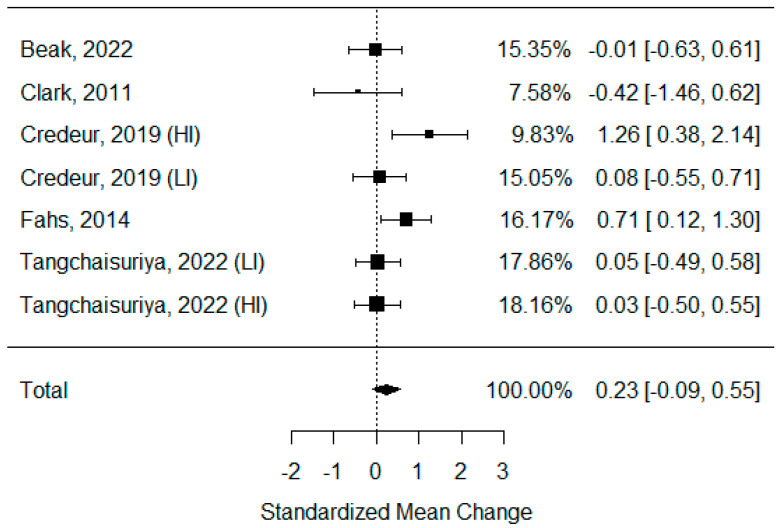
Forest plot describing the effects of BFR exercise on PWV values (LI—low-intensity BFR exercise, HI—high-intensity BFR exercise). Beak 2022 [3], Clark 2011 [49], Credeur 2019 [44], Fahs 2014 [50], Tangchaisuriya 2022 [39].

Six studies examining changes in PWV were identified, but one of them was not eligible for statistical analysis, due to insufficient data [43]. As some of the studies consisted of different subgroups performing different types of exercise, all variants were pooled, resulting in seven trials being pooled to examine the effects of exercise on the pulse wave velocity. The model estimate was 0.230 (CI −0.093, 0.554, *p* value = 0.163). The heterogeneity was moderate (I-squared: 41.87%, *p* value = 0.083) [Figure 3].

#### 3.3.4. Intima-Media Thickness

Only the study by Tangchaisuriya et al. [39] examined the IMT values. However, it did not present any significant changes after BFR exercise and showed no difference compared to high-intensity or low-intensity non-BFR training.

#### 3.3.5. Nitric Oxide

Boneo et al. [51] reported that the increase in NOx was more significant after BFR exercise than after high-intensity non-BFR exercise but did not differ from that following low-intensity non-BFR exercise. The other three studies showed no differences in the NO concentration between BFR and non-BFR training [29,38,52], but Remis et al. reported a significant elevation of NO after both forms of training [38]. The obtained data were insufficient for calculations.

### 3.4. Angiogenesis

#### 3.4.1. Vascular Endothelial Growth Factor and Its Variations

Fifteen trials analyzed vascular endothelial growth factor in multiple forms and its receptors: serum VEGF (*n* = 9), VEGF mRNA (*n* = 5), and VEGF-R (*n* = 6).

**Figure 4:**
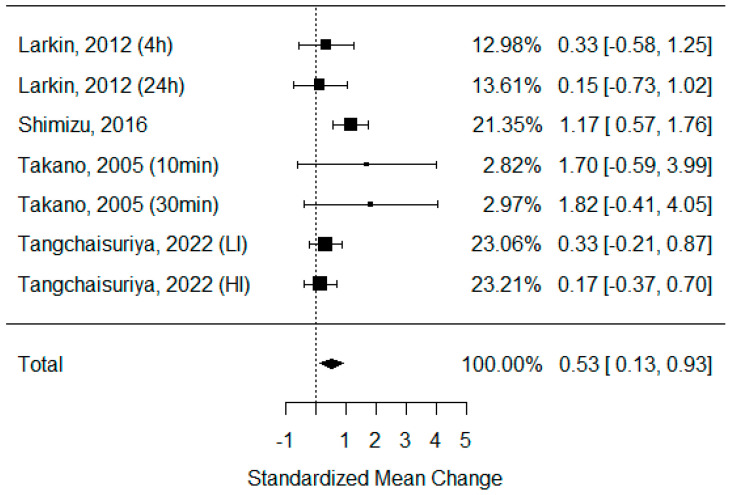
Forest plot describing the effects of BFR exercise on serum VEGF values (4 h—measured 4 h after exercise, 24 h—measured 24 h after exercise, 10 min—measured 10 min after exercise, 30 min—measured 30 min after exercise, LI—low-intensity BFR exercise, HI—high-intensity BFR exercise). Larkin 2012 [52], Shimizu 2016 [42], Takano 2005 [27], Tangchaisuriya 2022 [39].

- Serum VEGF: Only four studies that examined serum VEGF were eligible for statistical analysis [27,39,42,52]. The rest did not present sufficient data for calculation, but they showed a trend for higher VEGF concentrations after BFR compared to non-BFR exercise [2,53,54,55]. Only in the study by Christiansen et al. [56] were no differences observed. As some of the studies consisted of different subgroups performing different types of exercise or/and used assessments at multiple time points, all of the variants were pooled, resulting in seven trials to be examined regarding the effects of exercise on circulating VEGF concentrations. The model estimate was 0.529 (CI 0.130, 0.928, *p* value = 0.009). The heterogeneity was low (I-squared: 39.41%, *p* value = 0.130) [Figure 4].
- VEGF mRNA: Five studies analyzed levels of VEGF mRNA, and 4 showed a significant increase after BFR exercise and a greater effect compared to non-BFR exercise, regardless of the training type [52,57,58,59]. Only Conceicao et al., in their 2016 study, did not observe significant changes in VEGF mRNA concentrations [60]. Unfortunately, the provided data were insufficient for calculations.
- VEGF-R: The serum VEGF-R concentration was only measured by two studies, but they both confirmed its significant elevation after BFR exercise, which was greater compared to that after non-BFR exercise [28,55]. VEGF-R mRNA was assessed in three studies [52,58,59], and all of them confirmed its peak due to BFR exercise, but it was only significantly different compared to non-BFR exercise in two of them.

#### 3.4.2. CD31 (PECAM-1) and CD34

Only Maga et al. [28] analyzed the concentrations of CD31 and CD34. They both showed significant elevations after BFR exercise that were higher compared to those after non-BFR exercise. Montgomery et al. [61] examined CD34+CD45dim cells and showed that they did not significantly change in count after BFR exercise. They also assessed CD34+VEGFR2+ and CD34+CD45dimVEGFR2+; the counts of both changed after BFR exercise but were considerably lower than after non-BFR exercise.

#### 3.4.3. CD106/VCAM-1

We did not identify any study that analyzed concentrations of VCAM-1 that could be included in this systematic review.

#### 3.4.4. Von Willebrand Factor

According to Shimizu [42], the serum concentration of vWF decreased significantly after BFR exercise, but there was no difference compared to that after non-BFR exercise.

### 3.5. Other Vascular Functions

#### 3.5.1. Ankle-Brachial Index and Toe-Brachial Index

**Figure 5:**
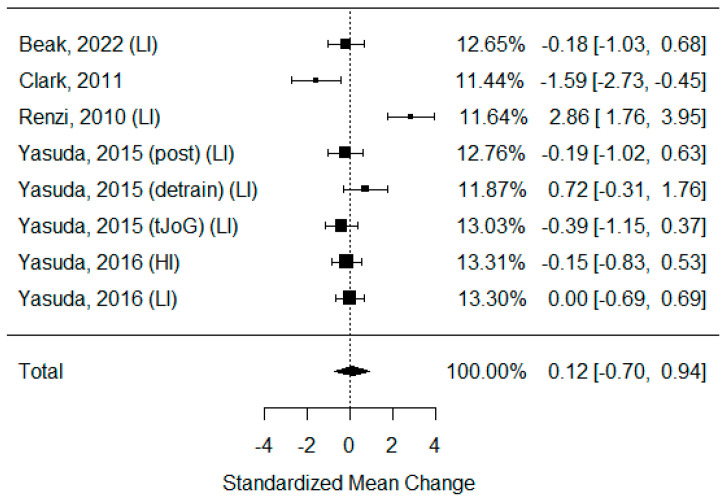
Forest plot describing the effects of BFR exercise on ABI values (post—measured immediately after exercise, detrain—measured up to 1 h after exercise, LI—low-intensity BFR exercise, HI—high-intensity BFR exercise). Beak 2022 [3], Clark 2011 [49], Renzi 2010 [34], Yasuda 2015 (post/detrain) [40], Yasuda 2015 (tJoG) [41], Yasuda 2016 [46].

Six studies that examined changes in the ABI were identified [3,34,40,41,46,49]. As some of the studies consisted of different subgroups performing different types of exercise, all variants were pooled, resulting in eight trials being pooled to examine the effects of exercise on the ankle-brachial index. None of the publications presented any significant changes after BFR exercise. The model estimate was 0.119 (CI −0.703, 0.941, *p* value = 0.776). The heterogeneity was high (I-squared: 86.55%, *p* value < 0.001) [Figure 5]. 

None of the studies enrolled in this systematic review assessed the toe-brachial index.

#### 3.5.2. Cardio-Ankle Vascular Index

Only six studies analyzed the impact of BFR exercise on the CAVI [5,35,40,41,46], but the data provided were insufficient for calculations. Only one of them presented a significant reduction in CAVI after BFR exercise [35]. However, none of the studies showed significant differences between BFR and non-BFR exercise regarding CAVI reduction, regardless of the activity type or intensity.

#### 3.5.3. TcPO2

Only Shimizu et al. [42] analyzed the impact of BFR exercise on TcPO2. The results suggest an increase in oxygen pressure after BFR exercise, but this was not different from that after non-BFR activity.

#### 3.5.4. Systolic Blood Pressure

**Figure 6:**
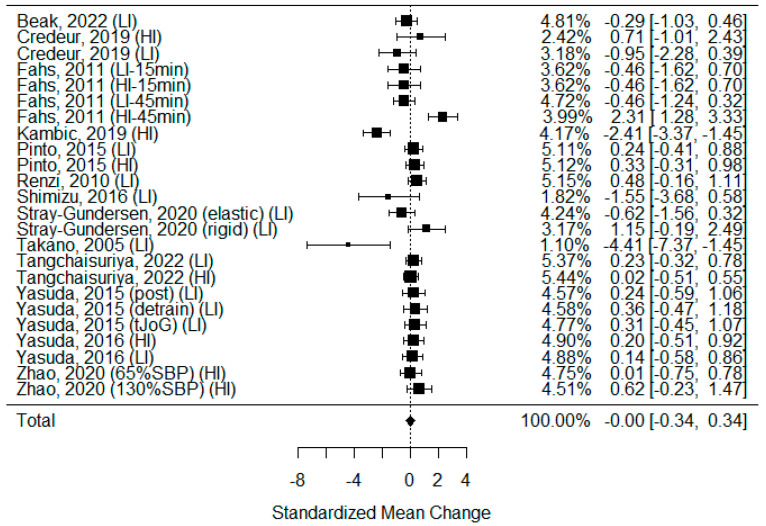
Forest plot describing the effects of BFR exercise on systolic blood pressure values (LI—low-intensity BFR exercise, HI—high-intensity BFR exercise, 15 min—measured 15 min after exercise, 45 min—measured 45 min after exercise, elastic—elastic band used for BFR induction, rigid—rigid band used for BFR induction, 65%SBP—pressure in BFR cuffs equals 65% of the systolic blood pressure, 130%SBP—pressure in BFR cuffs equals 130% of the systolic blood pressure). Beak 2022 [3], Credeur 2019 [44], Fahs 2011 [48], Kambic 2019 [31], Pinto 2015 [30], Renzi 2010 [34], Shimizu 2016 [42], Stray-Gundersen 2020 [35], Takano 2005 [27], Tangchaisuriya 2022 [39], Yasuda 2015 (post/detrain) [40], Yasuda 2015 (tJoG) [41], Yasuda 2016 [46], Zhao 2020 [55].

Among the studies included in the systematic review, 20 examined changes in systolic blood pressure after BFR and non-BFR exercise [3,5,27,29,30,31,34,35,36,39,40,41,42,43,44,45,46,47,48,55], of which 14 were eligible for calculations. As some of the studies consisted of different subgroups performing different types of exercise or/and used assessments at multiple time points, all variants were pooled, resulting in 24 trials being examined to determine the effects of exercise on SBP. The model estimate was −0.002 (CI −0.343, 0.339; *p* value = 0.990). The heterogeneity was present in all studies (I-squared: 72.08%, *p* value < 0.001). In eight studies, SBP increased significantly after BFR exercise [5,27,29,35,42,44,45,62], while in two, the value decreased significantly [31,36]. Only in four studies was the elevation of SBP more significant after BFR than after non-BFR exercise [27,30,35,42] [Figure 6].

#### 3.5.5. Heart Rate

**Figure 7:**
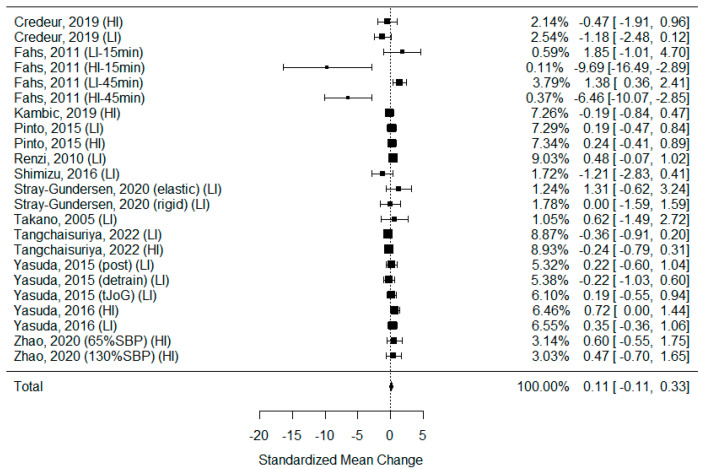
Forest plot describing the effects of BFR exercise on heart rate values (LI—low-intensity BFR exercise, HI—high-intensity BFR exercise, 15 min—measured 15 min after exercise, 45 min—measured 45 min after exercise, elastic—elastic band used for BFR induction, rigid—rigid band used for BFR induction, 65%SBP—pressure in BFR cuffs equals 65% of the systolic blood pressure, 130%SBP—pressure in BFR cuffs equals 130% of the systolic blood pressure). Credeur 2019 [44], Fahs 2011 [48], Kambic 2019 [31], Pinto 2015 [30], Renzi 2010 [34], Shimizu 2016 [42], Stray-Gundersen 2020 [35], Takano 2005 [27], Tangchaisuriya 2022 [39], Yasuda 2015 (post/detrain) [40], Yasuda 2015 (tJoG) [41], Yasuda 2016 [46], Zhao 2020 [55].

Eighteen studies examining heart rate changes after BFR and non-BFR exercise were included in this systematic review [5,27,29,30,31,34,35,36,39,40,41,42,44,45,46,47,48,55]. Thirteen of them were eligible for calculations. As some of the studies consisted of different subgroups performing different types of exercise or/and involved assessments at multiple time points, all variants were pooled, resulting in 23 trials being examined to determine the effects of exercise on HR. The model estimate was 0.113 (CI −0.109, 0.335, *p* value = 0.319). The heterogeneity was moderate (I-squared: 24.34%, *p* value < 0.001) [Figure 7].

In nine studies, a significant elevation of HR was observed after BFR exercise [5,27,29,35,42,44,45,47,48], while in only a single study, the value decreased significantly [55]. In five studies, the elevation of HR was more significant after BFR compared to that after non-BFR exercise [27,29,30,35,42]. According to Fahs et al. [48], the elevation of HR was less intense after BFR than after non-BFR exercise but only compared to high-intensity training.

## 4. Discussion

### 4.1. Impact of Blood Flow Restriction on Exercise Performance

Improvements in exercise techniques remain one of the greatest challenges of physiotherapy and sports medicine. With increasing expectations for better performance, there is a growing expectation that training methods should be more efficient. Modern training should provide the same or even better results with a shorter exercise time and with less physical effort. BFR training was meant to be one of the answers to this urgent matter [63,64]. There is an extensive number of publications demonstrating increases in muscle hypertrophy and strength through resistance exercise combined with BFR exercise. Numerous systematic reviews and meta-analyses have illustrated the effectiveness of this combination for enhancing skeletal muscle strength, including dynamic isotonic, isometric, and isokinetic strength, as well as the rate of force development strength capacity [65,66]. It is also well established that muscle hypertrophy and strength adaptations achieved by resistance exercise with BFR exercise are generally more significant than those achieved with resistance exercise with a low load alone. They are comparable with the increases in strength observed following high-load resistance exercise [67]. Improvements are observed in a relatively short period, even after 2–10 days of training [68,69], which can also be the result of a higher exercise frequency, which may be impossible with high-load training, where recovery time is prolonged. In the case of regular low-load resistance training, the time needed to achieve such results is much longer, from 3 up to even 8 weeks [46,70,71].

On the contrary, the BFR method is still a matter of controversy [72,73,74]. The variety in BFR devices leads to confusion about the restriction of blood flow with total arterial occlusion. Not all types of training, especially high-load resistance exercise, should be performed in complete ischemia. In addition, too much compression of the veins can cause increased venous pressure. It can damage vein valves and ultimately result in chronic venous insufficiency [75]. Furthermore, the elevation of the mean systolic arterial blood pressure during and after BFR sessions (Figure 6) raises concerns, as it is a decisive risk factor for potential cardiovascular adverse events.

### 4.2. Other BFR Vascular-Related Studies

At first, reviews similar to ours were looked for, and three publications were identified, but none covered our entire search area and were up-to-date [76,77,78]. Pereira-Neto et al. excluded a number of articles that met the inclusion criteria set by them [76]. Furthermore, the analysis methodology was questionable, as the studies were grouped by the type of vascular function assessed, even though multiple outcomes were measured in different ways yet pulled into the same analysis. In our opinion, this could have distorted the results, leading to far-reaching conclusions and assumptions. Li et al. focused on angiogenesis-related factors but were limited only to skeletal muscles, while circulating pro- and anti-angiogenesis factors were not analyzed [77]. On the other hand, the analysis conducted by Liu et al. included a broad spectrum of vascular outcomes but only assessed them after resistance BFR exercise [78]. This means that numerous other types of exercise combined with the BFR method were excluded, and their impacts on vascular functions remain unanalyzed.

### 4.3. Vascular Parameters

Although physical training is widely recognized to have positive impacts on endothelial functions, specific effects on endothelial vasodilation abilities are still uncertain, particularly regarding an increase in FMD and a reduction in vascular stiffness, depending on the intensity and type of training, and the optimal balance between these factors and endothelial responses [79]. A complex assessment of vascular properties should include endothelial functions and angiogenesis. The combination of these two groups of parameters characterizes the performance of the main vessels and the whole process of creating new vasculature and expanding the arterial network. Additionally, clinical hemodynamic parameters, such as systolic/diastolic blood pressure or heart rate, do not directly reflect either of these categories but give critical clinical data on the condition of the whole cardiovascular system. Finally, there are microcirculation parameters, such as TcPO2, that also need to be mentioned in the analysis of vascular parameters.

#### 4.3.1. Endothelium

The parameter of the endothelium most commonly used in clinical practice is the IMT. This describes the thickness of the intima-media layer of the carotid arteries. Even though its enlargement is no longer interpreted as a subclinical phase of atherosclerosis, it is still recognized as an increased cardiovascular risk factor [80,81,82]. Only one single study included in this systematic review analyzed this parameter. In the young and healthy population, which in most cases were the subjects of the studies, the IMT is mainly within the normal range, so there is no room for improvement. Furthermore, the observation time was probably not long enough to observe the changes in the thickness of the intima-media.

From a physiological point of view, the most popular endothelial function assessment is FMD. This index is based on the difference between the diameter of the artery before, during, and after the release of brachial artery occlusion [83]. An increased FMD represents an improvement in the vasodilatory functions of the arterial wall, leading to better pressure control and greater oxygenation of tissue and organ oxygenation [84]. This phenomenon has also been confirmed with other forms of training [85]. Our results show that BFR exercise is the type of exercise that also stimulates changes in vasodilatation, improving blood flow within the main arteries. Furthermore, BFR exercise improves FMD more significantly than regular activity (Figure 2). However, due to the large heterogeneity index, we must treat this result with caution.

The reactive hyperemia index is also used to assess endothelial vasodilatation but within the microvascular system [86]. It is strictly related to atherosclerosis-based cardiovascular disease [87]. Its noninvasive characteristic and high sensitivity make this method quite popular in the sports and rehabilitation field. According to Higashi et al., even daily aerobics significantly improves vasodilatation within microvessels [88]. Surprisingly, we found only a few studies that examined the impact of BFR exercise on this parameter, which did not produce any conclusive results [28,39,42]. This could again be due to the characteristics of the population, who were mostly young and healthy with generally normal baseline RHI values.

Vascular stiffness is a physical phenomenon that can be defined by multiple parameters. Its reduction generally promotes the reduction in blood pressure and reduces the risk of atherosclerosis [89]. In this systematic review, we focused on the most commonly used parameters, but only the pulse wave velocity data were homogeneous enough and had sufficient quality to be included in the meta-analysis [3,39,44,49,50]. Unfortunately, this parameter did not yield any conclusive results. There were also no differences between BFR and non-BFR activities in the augmentation index or its corrected heart rate version (AI75), except in the study conducted by Amorim et al. [43]. However, Karabulut et al. confirmed that the SVR was reduced more efficiently after BFR exercise, although this was highly dependent on the intensity of the exercise performed [47].

Nitric oxide is the primary biochemical biomarker of endothelial vasodilation. It is responsible for vasodilation regulation and is crucial for arteries’ adaptation to stress or sports activities [90]. Physical activities generally account for its elevation not only in terms of an acute reaction but also in the form of a long-term outcome of regular exercise [91]. This is in opposition to the results of our analysis, as only one study observed the elevation of NO [38]. We hypothesize that this could be due to the analyzed studies’ questionable methodologies, as blood samples were taken immediately after exercise. Furthermore, two studies were based only on single sessions [29,52]. Additionally, in the study conducted by Barilli et al., the participants were suffering from hypertension, so we assume that the production of endothelial NO in this population was already impaired by comorbidities [29].

#### 4.3.2. Angiogenesis

Physical activity is one of the crucial factors for the stimulation of angiogenesis, even after short-term exercise, but it is performed frequently [92]. During physical exercise, especially in the anaerobic phase, the production of proangiogenic factors, such as VEGF, soluble endoglin, hypoxia-inducible factor 1 (HIF-1), and peroxisome proliferator-activated receptor gamma coactivator (PGC-1α), is stimulated [93,94]. This phenomenon is widely used in the rehabilitation of patients with intermittent claudication during the course of peripheral arterial disease [95,96]. As a result, pain reduction and extension of the claudication distance are observed, combined with the progression of wound healing in patients with chronic limb-threatening ischemia [97].

The matter of BFR training’s influence on angiogenesis has been poorly examined. So far, only one meta-analysis has been performed, and this only analyzed the muscle concentrations of angiogenic factors [77]. A few of the included articles were not eligible for our study, as they did not meet the methodology inclusion criteria, i.e., blood flow restriction applied after but not during training [98] or blood flow restriction induced by gravity but not with any restriction tool [99].

Our results are mostly significant for evaluating changes in the VEGF concentration. Most of the studies included in this analysis confirmed that BFR exercise induces VEGF production. The meta-analysis showed that exercise with the restriction of blood flow is more effective for stimulating angiogenesis than regular activity (Figure 4), but the confidence interval was not far from “0”, and after a data sensitivity subgroup analysis, the result could be different. Additionally, increased expression of VEGF mRNA was observed after BFR training, and in all cases, the elevation was more significant after BFR training compared to regular training [52,57,58,59]. We only found three studies that examined angiogenic factors other than VEGF. Shimizu et al. showed a decrease in the von Willebrand Factor, but this was comparable to that after non-BFR exercise. PECAM-1 and CD-34 concentrations increased significantly more than after exercise without BFR exercise [28], but CD34+CD45dim, CD34+VEGFR2+, and CD34+CD45dim VEGFR2+ did not show a superiority in count after BFR vs. non-BFR exercise [61].

#### 4.3.3. Other Vascular Functions

The ankle-brachial index is the most basic and yet the most commonly used lower-extremity ischemia diagnosis method [16]. Stenosis of peripheral artery occlusion leads to a reduction in the peripheral arterial pressure, which causes ischemia of the muscles and skin. This index remains one of the most important scores for assessing therapy outcomes [100]. In this analysis, we did not show a significant improvement in ABI after BFR training or its superiority over non-BFR exercise. It should be emphasized that, in most studies, participants were relatively young and healthy, and the baseline ABI results were already within the normal range. Studies in patients with impaired peripheral circulation are needed to assess the real impact of BFR exercise on changes in ABI. The same lack of improvement was observed for the CAVI score, and we hypothesize that the reason for this was the same. TcPO2 was examined only in one study with a small sample size. Its result suggests that it is stimulated similarly regardless of the exercise type, but it improved compared to the baseline level. The results are inconclusive, and further studies are needed to examine this phenomenon.

Systolic blood pressure and heart rate are variables that describe the dynamics of arterial blood flow. During physical activity, the heart rate increases to supply the muscles and brain with additional oxygen-rich blood. After activity, the HR decreases, and in people who regularly perform sport, it is lowered at rest [101]. The frequent performance of physical activity also reduces blood pressure [102]. The European Society of Cardiology recommends it as one of the elements of hypertension treatment and rehabilitation after myocardial infarction [103]. The results of this meta-analysis did not confirm those tendencies. This could be due to the short time between the completion of the training and measurement, which occurred in most of the included studies. Although there was a trend for an elevated heart rate after exercise, it was not statistically significant.

### 4.4. Study Limitations

A limited number of randomized clinical trials have studied the impact of BFR training on vascular functions. Most studies are underpowered, which can influence the results obtained. To overcome this issue, we decided to treat the comparison of different groups as separate studies. This solution also has a drawback—the same population is included multiple times and may lead to bias in the population characteristics. Another issue is the lack of standardization in activity protocols: the length of exercise in the studies included in this analysis varied from single sessions to five sessions per week for 8 weeks [55]. There are multiple studies that indicate that acute improvements in endothelial functions after exercise are temporary and long-term training is needed to obtain a permanent effect [104,105]. Another factor is the difference in the type of exercise, as there are studies indicating that the type of exercise is a critical factor for changes in endothelial functions [106]. This also influences the activation of angiogenesis [107]. Additionally, the difference in the degree of blood flow restriction is not without significance and could lead to bias in the analysis. All of these differences reduce the comparability of these studies, and the results of this meta-analysis must be taken with great caution. Finally, the bias analysis revealed that almost none of the studies were blinded for participants or researchers (Supplementary Figure S1). This is a common problem in experimental studies involving physical therapy, as it is difficult or even impossible to blind the form of intervention when it is an activity. Even if patients are not told which branch is included, they can easily estimate the type of activity they are performing [108]. Most of the studies analyzed did not involve long-term observations after finishing the training program (Supplementary Figure S1). This limits our conclusions on the durability of the effect of BFR exercise on the measured vascular parameters. It should also be mentioned that three studies included in the analysis did not have a typical control group—the comparison was made between different extremities but within the same patients [32,37,50]. This is a significant methodological issue, as the endothelium is currently considered a single endocrine organ [109]. This implies that even local stimulation impacts the whole endothelium in a similar way. Despite this, we decided to include those studies due to the already low number of other randomized clinical trials and typical crossover studies.

## 5. Conclusions

The results obtained current trends that suggest the significant impact of BFR training on endothelial functions and angiogenesis. Mainly, arterial vasodilation functions and VEGF-based angiogenesis seem to be influenced more by BFR training than by regular activity without the restriction of blood flow. There is still a lack of multicenter randomized clinical trials that include large numbers of participants. More studies, addressing not only young, healthy subjects, are needed to confirm the advantage of BFR over non-BFR activity in terms of arterial function and angiogenesis stimulation.

## Data Availability

Not applicable.

## Supplementary Materials

The following supporting information can be downloaded at: https://www.mdpi.com/article/10.3390/biomedicines11061601/s1, Figure S1: Graphs of risk of bias in accordance with the Joanna Briggs Institute guidelines; Table S1: Data extraction table.

## Appendix A

**Table A1:** The detailed search strategy.

PUBMED:
((“blood flow restricted training”) OR (“blood flow restriction training”) OR (“blood flow restricted exercise”) OR (“blood flow restriction exercise”) OR (“blood flow restriction”) OR (BFR) OR (“BFR-RT”) OR (“ischemic training”) OR (“ischemic exercise”) OR (kaatsu) OR (katsu) OR (“kaatsu exercise”) OR (“kaatsu training”) OR (“vascular occlusion exercise”) OR (“vascular occlusion training”)) AND ((“vascular function”) OR (“endothelium”[MeSH Terms]) OR (“endothelium, vascular”[MeSH Terms]) OR (vasodilatation[MeSH Terms]) OR (“flow mediated dilatation”) OR (“flow-mediated dilatation”) OR (FMD) OR (“reactive hyperemia index”) OR (RHI) OR (“intima-media thickness”) OR (IMT) OR (“transcutaneous oximetry”) OR (tcpo2) OR (“arterial stiffness”) OR (“ankle brachial index”[MeSH Terms]) OR (“toe brachial index”) OR (“toe- brachial index”) OR (TBI) OR (“cardio ankle vascular index”[MeSH Terms]) OR (“vascular endothelial growth factor a”[MeSH Terms]) OR (“vascular endothelial growth factor b”[MeSH Terms]) OR (vascular stiffness[MeSH Terms]) OR (“vascular remodeling”[MeSH Terms]) OR (Angiogenesis effect[MeSH Terms]) OR (angiogenesis factor[MeSH Terms]) OR (“angiogenesis inducing agents”[MeSH Terms]) OR (angiogenesis) OR (cd31 antigen[MeSH Terms]) OR (vcam-1) OR (CD106) OR (CD-106) OR (von willebrand factor[MeSH Terms]) OR “nitric oxide”[MeSH Terms]))
EMBASE:
(‘blood flow restriction training’/de OR ‘blood flow restriction exercise’/de OR ‘blood flow restriction’/de OR ‘ischemic training’ OR ‘ischemic exercise’ OR ‘kaatsu’ OR ‘katsu’ OR ‘kaatsu training’ OR ‘kaatsu exercise’ OR ‘blood vessel occlusion’/de OR ‘vascular occlusion exercise’ OR ‘vascular occlusion training’) AND (‘vascular function’/de OR ‘endothelium’/de OR ‘vascular endothelium’/de OR ‘vasodilatation’/de OR ‘flow mediated dilatation’/de OR ‘flow-mediated dilation test’/de OR ‘flow mediated vasodilation’/de OR ‘reactive hyperemia index’/de OR ‘arterial wall thickness’/de OR ‘intima-media thickness’ OR ‘transcutaneous oximetry’/de OR tcpo2 OR ‘ankle brachial index’/de OR ‘toe brachial index’/de OR ‘cardio-ankle vascular index’/de OR ‘vasculotropin’/de OR ‘vascular endothelial growth factor’ OR ‘arterial stiffness’/de OR ‘vascular remodeling’/de OR ‘angiogenesis’/de OR ‘angiogenic factor’/de OR ‘angiogenesis modulator’/de OR ‘cd31 antibody’/de OR ‘platelet endothelial cell adhesion molecule 1’/de OR ‘vascular cell adhesion molecule 1’/de OR ‘von willebrand factor’/de OR ‘nitric oxide’/de) AND [english]/lim AND ‘human’/de
