# Supplementary material for: Impact of Blood-Flow-Restricted Training on Arterial Functions and Angiogenesis—A Systematic Review with Meta-Analysis
Source: Biomedicines. 2023 May 31;11(6):1601. doi: 10.3390/biomedicines11061601 (PMC10295844; doi:10.3390/biomedicines11061601)

Supplemental Figure S1. Graphs of risk of bias in accordance to Joanna Briggs Institute guidelines.

A. Risk of bias in randomized control studies (n=13)

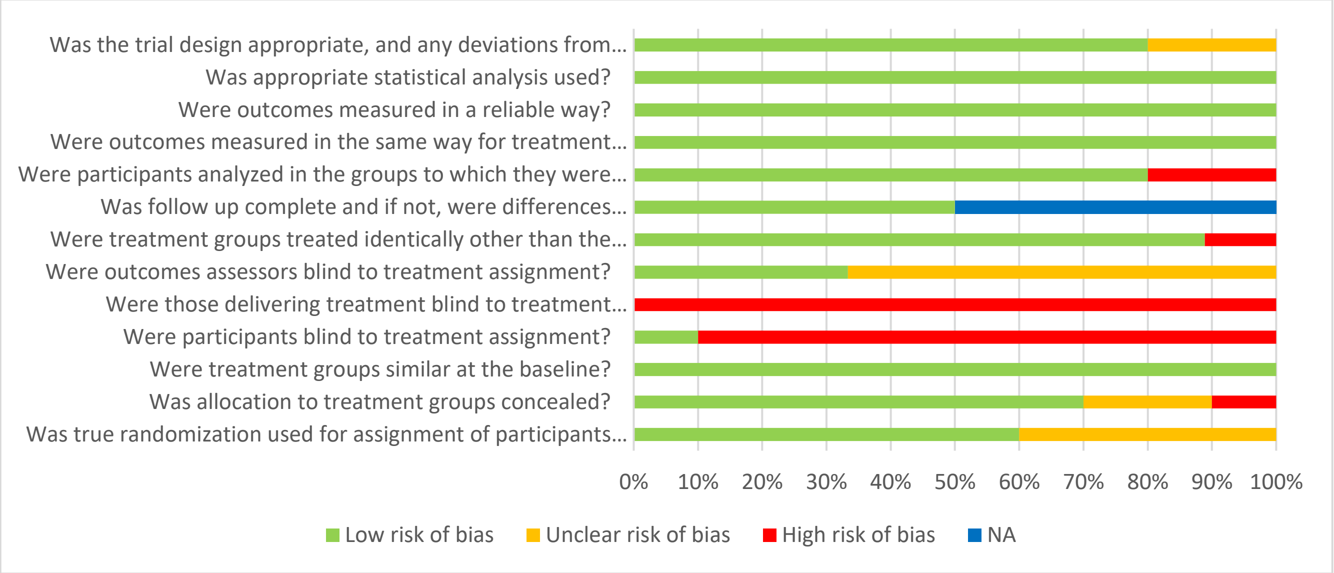

B. Risk of bias in cross-over and cross-over-like studies (n=25)

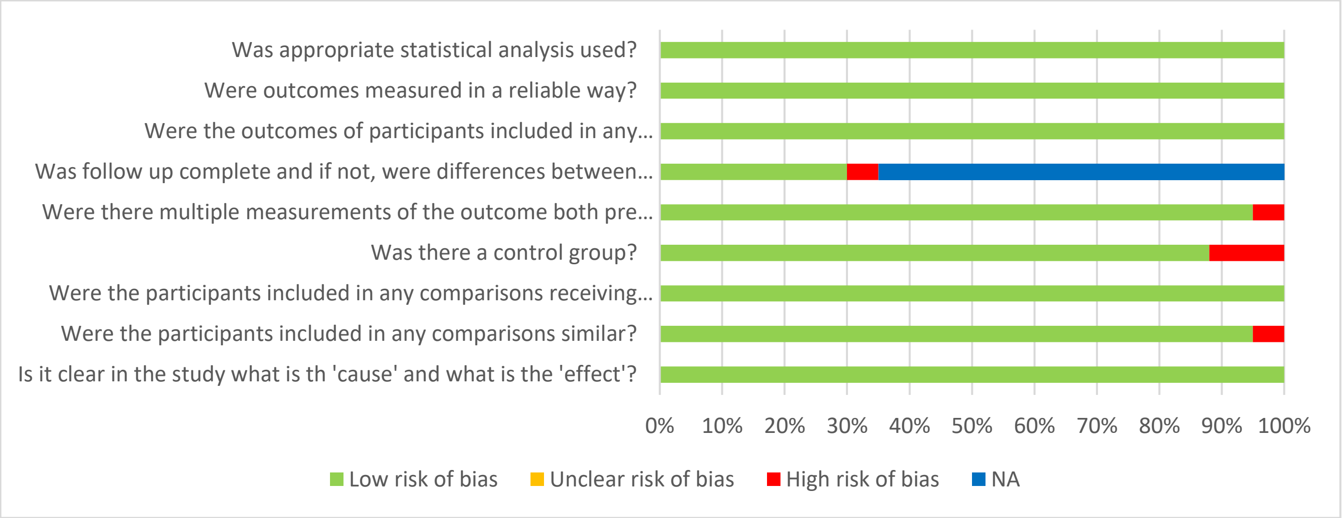

Supplement: Supplementary file 1 [file biomedicines-11-01601-s001.zip › Supplementary Figure S1.pdf]
