# Supplementary material for: Impact of Blood-Flow-Restricted Training on Arterial Functions and Angiogenesis—A Systematic Review with Meta-Analysis
Source: Biomedicines. 2023 May 31;11(6):1601. doi: 10.3390/biomedicines11061601 (PMC10295844; doi:10.3390/biomedicines11061601)
Supplement: Supplementary file 1 [file biomedicines-11-01601-s001.zip › Supplementary Table S1 - data extraction.pdf]

Number	Authors	Title	Year	Journal	Volume, pages	Country of the study	Type of the study	Total number of participants	Number of participants in study group	Male [%]	Mean age [years +/-SD]	Population type	Type of the exercise	Length of exercise [number of sessions; number of weeks]	Duration of 1 session [minutes]	Scheme of 1 session [number of repeats]	Blood flow restriction time [minutes]	Blood flow restriction type	Name of the BFR tool	Pressure used for BFR [mmHg]	Control group training	Which vascular and angiogenesis function parameters were assessed?	Diffrences between after BFR and nonBFR groups in vascular parameters	Diffrences between pre-BFR and after-BFR in vascular parameters	Diffrences between after BFR and nonBFR groups in blood pressure	Diffrences between pre-BFR and after-BFR in blood pressure and heart rate
1	Amorim S. et al.	The Effect of a Single Bout of Resistance Exercise with Blood Flow Restriction on Arterial Stiffness in Older People with Slow Gait Speed: A Pilot Randomized Study.	2022	Journal of Cardiovascular Development and	9(3):85	Brazil	RCT	17	10	17,60%	84.47	healthy, older	resisetance exercise	1 single session	N/A	3 sets of 15 repeats (20% 1RM)	N/A (during whole training)	pressure cuff	Kaatsu Nano	150-260mmHg	Same but high resistance	PWV<br>AI<br>AI75	BFR=nonBFR<br>**BFR**>nonBFR<br>BFR=nonBFR	-<br>=<br>=	SBP: BFR = nonBFR	+
2	Barili A. et al.	Acute responses of hemodynamic and oxidative stress parameters to aerobic exercise with blood flow restriction in hypertensive elderly women	2018	Molecular Biology Reports	45(5):1099-1109	Brazil	cross-over study	16	16	0%	67.2 ± 3.7	hypertensive women	treadmill exercise	N/D	N/D	N/D	N/D	pressure cuff	regular sphygmomanometer cuffs	130% resting SBP	Same low and high intensity	NOx	N/D	=	SBP: BFR = nonBFR<br>SBP: BFR = nonBFR HI<br>HR: BFR>nonBFR<br>HR: BFR=nonBFR HI	+<br>+
3	Barjaste A. et al.	Concomitant aerobic- and hypertrophy-related skeletal muscle cell signaling following blood flow-restricted walking	2021	Science & Sports	10.1016/j.scispo.2020.03.00(	Iran / France	cross-over study	5	5	100%	33.41 ± 1.02	healthy, young	walking	1 single session	10	N/A	14	pressure cuff	Kaatsu-Master system	200 mmHg	Same training	VEGF	**BFR**>nonBFR	=	N/A	N/A
4	Beak H.J. et al.	Effect of Low-Intensity Aerobic Training Combined with Blood Flow Restriction on Body Composition, Physical Fitness, and Vascular Responses in Recreational	2022	Healthcare	10(9):1789	South Korea	RCT	29	14	100%	29.93 ± 3.96	healthy, young, amateur sportsmen	treadmill exercise	3/week; 8 weeks	15	10 minutes warm-up, 5*2 min running + 1min rest (40% VO2max)	N/D	pressure cuff	Kaatsu Nano	160-240mmHg	Same training	FMD<br>PWV<br>ABI	BFR=nonBFR<br>BFR=nonBFR<br>BFR=nonBFR	N/D<br>N/D<br>N/D	SBP: BFR = nonBFR	N/D
5	Boeno F. P. et al.	Acute effects of strength exercise with blood flow restriction on vascular function of young healthy males.	2018	Jornal Vascular Brasileiro	17(2): 122–127	Brazil	cross-over study	11	11	100%	23.72±3.49	healthy, young	resistance exercise	1 single session	N/A	Repeats up to fatigue of arms/legs	N/A (during whole training)	pressure cuff	regular sphygmomanometer	P -20mmHg; legs: SBP +	Same low and high intensity	NOx	BFR=nonBFR<br>**BFR**>nonBFR HI	=	N/A	N/A
6	Christiansen D. et al.	Blood flow-restricted training enhances thigh glucose uptake during exercise and muscle antioxidant function in humans	2019	Metabolism: Clinical and Experimental	98:1-15	Denmark	n-RCT - experimental study	13	13	100%	25 ± 4.0	healthy, young	cycling ergometer	1 single session	34	5min warm-up, 9*2min high performance, 2*5min + 6*1min rest without resistance	18	pressure cuff	regular sphygmomanometer cuffs	180mmHg	Same training on the other leg	VEGF	BFR=nonBFR	+	N/A	N/A
7	Clark B. C. et al.	Relative safety of 4 weeks of blood flow-restricted resistance exercise in young, healthy adults.	2011	Scandinavian Journal of Medicine &	21: 653–662	USA	RCT	16	9	88%	23.96 ± 1.58	healthy, young	resistance exercise	3/week; 4 weeks	N/A	3 series, 8-12 repeats each (30% 1RM)	N/A (during whole training)	pressure cuff	E20 Rapid Cuff Inflator	130% resting SBP	Same training but 80% 1RM	ABI<br>PWV	BFR=nonBFR<br>BFR=nonBFR	=<br>=	N/A	N/A
8	Conceição M. S. et al.	Augmented Anabolic Responses after 8-wk Cycling with Blood Flow Restriction.	2019	Medicine and Science in Sports and Exercise	51(1):84-93	Brazil	RCT	30	10	100%	22.67 ± 2.67	healthy, young	cycling ergometer	1/week; 8 weeks	30 (40% VO2)	N/A	30	pressure cuff	regular sphygmomanometer cuffs	80% resting SBP	Same endurance training but 70% VO2 or Resistance training (70% 1RM on leg press exercise - 4 sets of maximum repetitions)	VEGF mRNA	**BFR**>nonBFR	+	N/A	N/A
9	Conceição M. S. et al.	Attenuated PGC-1α Isoforms following Endurance Exercise with Blood Flow Restriction.	2016	Medicine and Science in Sports and Exercise	48(9):1699-707	Brazil	cross-over study	9	9	100%	22.4 ± 3.0	healthy, young	cycling ergometer	1 single session	15 (40% VO2)	N/A	15	pressure cuff	regular sphygmomanometer cuffs	80% resting SBP	Same endurance training but 70% VO2 for 30 minutes or Resistance training (70% 1RM on leg press exercise - 4 sets of 10 repetitions)	VEGF mRNA	BFR<**nonBFR**	=	N/A	N/A
10	Credeur D. P. et al.	Effects of handgrip training with venous restriction on brachial artery vasodilation.	2010	Medicine and Science in Sports and Exercise	42(7):1296-302	USA	cross-over-like study	12	12	41,67%	22 ± 1.0	healthy, young	handgrip + forearm contraction	3/week; 4 weeks	20	15 repeats / 1 minute	20	pressure cuff	regular sphygmomanometer cuffs	80mmHg	Same training	FMD	BFR<**nonBFR**	-	N/A	N/A
11	Credeur D. P. et al.	Central cardiovascular hemodynamic response to unilateral handgrip exercise with blood flow restriction.	2019	European Journal of Applied Physiology	119(10):2255-2263	USA	cross-over study	15	15	100%	25 ± 2	healthy, young	handgrip	1 single session	(40% and 60% MV	20 repeats / 1 minute	5	pressure cuff	E20 Rapid Cuff Inflator	80-100mmHg	Same training with 60% MVC	PWV<br>AI<br>RM	BFR HI=nonBFR<br>BFR LI=nonBFR<br>BFR HI=nonBFR<br>BFR LI=nonBFR<br>BFR HI=nonBFR<br>BFR LI=nonBFR	+<br>=<br>=<br>=<br>=<br>=	HR: BFR HI=nonBFR HI<br>HR: BFR LI=nonBFR HI<br>SBP: BFR HI=nonBFR HI<br>SBP: BFR LI=nonBFR HI	+<br>=<br>+<br>+
12	Early K.S. et al.	Effect of blood flow restriction training on mascular performance, pain and vascular function	2020	International Journal of Sports Physical	15(6):892-900	USA	RCT	21	11	38,10%	23.5 ± 4	healthy, young	resistance exercise	20 session (2-3/week, 8 weeks)	N/A	10-15 min warm up, 5 exercise: 3 sets of 30	N/A (during whole training)	elastic, pneumatic band	BStrong Training SystemsTM	250mmHg (arms), 350mmHg (legs)	Same but high resistance (60% 1RM)	FMD	BFR LI=nonBFR HI	+	HR: BFR LI=nonBFR HI<br>SBP: BFR LI=nonBFR HI	N/D<br>-
13	Fahs C. A. et al.	Effect of different types of resistance exercise on arterial compliance and calf blood flow	2011	European Journal of Applied Physiology	111:2969–2975	USA	cross-over study	11	11	100%	28 ± 5	healthy, young	resistance exercise	1 single session	N/A	1 series of 30 repeats + 3 series of 15 repeats (20% 1RM)	N/A (during whole training)	pressure cuff	Kaatsu-Master system	200mmHg	LI: 1 series of 30 repetitions + 3 series of 15 repetitions (20% 1RM)<br>HI: 3 series of 10 repetitions (70% 1RM)	SVR<br>LAEI<br>SAEI	BFR =nonBFR LI<br>BFR =nonBFR HI<br>BFR <nonBFR LI<br>**BFR** >nonBFR HI<br>BFR =nonBFR LI<br>BFR =nonBFR HI<br>BFR =nonBFR LI<br>BFR =nonBFR HI<br>BFR =nonBFR LI<br>BFR <**nonBFR HI**<br>BFR =nonBFR LI<br>BFR <**nonBFR HI**	-<br>=<br>+<br>=<br>-<br>=	ΔHR: BFR LI=nonBFR LI<br>ΔHR: BFR LI<nonBFR HI<br>ΔSBP: BFR LI=nonBFR LI<br>ΔSBP: BFR LI=nonBFR HI	+<br>+<br>=<br>=
14	Fahs C. A. et al.	Vascular adaptations to low-load resistance training with and without blood flow restriction.	2014	European Journal of Applied Physiology	114:715–724	USA	cross-over-like study	16	16	68,80%	55 ± 7	healthy, midage	resistance exercise	3/week; 6 weeks	N/A (30% 1RM)	2 sets of 20 repeats / 1 minute for 1-2 weeks, 3-4 same sets for 3-4 weeks, 5-6 same sets for 5-6 weeks	N/A (during whole training + 15 sec)	pressure cuff	KaatsuMaster-mini system	150mmHg or <50%A(	Same training on the other leg	PWV	BFR=nonBFR	+	N/A	N/A
15	Ferguson R. A. et al.	The acute angiogenic signalling response to low-load resistance exercise with blood flow restriction	2018	European Journal of Sport Science	18(3):397-406	UK	cross-over study	6	6	100%	26 ± 2	healthy, young	resistance exercise	1 single session	N/A (20% 1RM)	4 sets of 30 repeats (20repeats/ 1 min)	N/A (during whole training + 15 sec)	pressure cuff	E20 Rapid Cuff Inflator	110mmHg	Same training	VEGF mRNA<br>VEGF-R2 mRNA<br>eNOS mRNA	**BFR**>nonBFR<br>**BFR**>nonBFR<br>BFR=nonBFR<br>BFR=nonBFR<br>BFR=nonBFR<br>BFR>nonBFR	+<br>+<br>=<br>+<br>+<br>=	N/A	N/A
16	Gustafsson T. et al.	VEGF-A splice variants and related receptor expression in human skeletal muscle following submaximal exercise.	2005	Journal of Applied Physiology	98:2137-2146	Sweden	cross-over study	9	9	100	22 (18-26)	healthy, young	resistance exercise	1 single session	45	60repeats / minute	45	pressure cuff	regular pressure cuff	50 mmHg	same training without BFR	VEGF-R1 mRNA<br>VEGF-R2 mRNA<br>VEGF-A mRNA	**ΔBFR**>ΔnonBFR<br>ΔBFR=ΔnonBFR<br>ΔBFR=ΔnonBFR<br>**ΔBFR**>ΔnonBFR<br>**BFR**>nonBFR<br>BFR=nonBFR	+<br>+<br>=<br>+<br>+<br>=	N/A	N/A
17	Hunt J. E. A. et al.	Brachial artery modifications to blood flow-restricted handgrip training and detraining.	2012	Journal of Applied Physiology	112:956-961	UK	cross-over-like study	9	9	100	26 ± 4	healthy, young	resistance exercise	12 sessions, 4 weeks	N/A	3 sets of 20 contractions / minute (40% 1RM)	N/A (during whole training)	pressure cuff	regular pressure cuff	80 mmHg	same training without BFR	FMD	BFR=nonBFR	=	N/A	N/A
18	Kambič T. et al.	Blood flow restriction resistance exercise improves muscle strength and hemodynamics, but not vascular	2019	Frontiers in Physiology	10:656	Slovenia	RCT	24	12	75	60.5 ± 2.4	CAD	resistance exercise	16 sessions, 8 weeks	N/A	3 sets (8, 10, 12 reps, +2 every session)	N/A (during whole training)	pressure cuff	regular pressure cuff	SBP + 15 to 20 mmHg	aerobic exercise training	FMD	BFR=nonBFR	=	HR: BFR=nonBFR<br>SBP: BFR=nonBFR	=<br>-
19	Karabulut U. et al.	Small arteries stay stiff for a longer period following vibration exercises in combination with blood flow restriction	2018	ClinicalPphysiology and Functional Imaging	doi: 10.1111/cpf.12516.	USA	cross-over study	8	8	100	22.6 ± 2.2	healthy, young	squats	2 sessions	N/A	8 sets of 45s	6 minutes	pressure cuff	Kaatsu-Master	144% SBP	same training without BFR	SAEI<br>LAEI<br>SVR	BFR=nonBFR<br>BFR<**nonBFR**<br>BFR=nonBFR<br>BFR=nonBFR<br>BFR=nonBFR<br>BFR=nonBFR	=<br>-<br>=<br>-<br>-<br>=	HR: BFR>nonBFR<br>SBP: BFR=nonBFR	+<br>=
													push-ups	2 sessions	N/A	10 sets of 60s	6	pressure cuff	Kaatsu-Master	100 mmHg	same training without BFR	SAEI<br>LAEI<br>SVR	BFR=nonBFR<br>BFR<**nonBFR**<br>BFR=nonBFR<br>BFR=nonBFR<br>BFR=nonBFR<br>BFR=nonBFR	=<br>-<br>=<br>-<br>-<br>=	HR: BFR>nonBFR<br>SBP: BFR=nonBFR	+<br>=
20	Larkin K. A. et al.	Blood flow restriction enhances post-resistance exercise angiogenic gene expression.	2012	Medicine and Science in Sports and Exercise	44:2077-2083	USA	cross-over study	6	6	50%	22 ± 1	healthy, young	resistance exercise	1 single session	N/A	10 sets of 12 repeats	N/A (during whole training)	pressure cuff	Kaatsu-Master Mini	220 mmHg	same training without BFR	Serum VEGF<br>Muscle VEGF<br>VEGF mRNA<br>VEGF-R2 mRNA<br>eNOS mRNA	BFR=nonBFR<br>BFR=nonBFR<br>BFR=nonBFR<br>BFR=nonBFR<br>**BFR**>nonBFR<br>**BFR**>nonBFR<br>**BFR**>nonBFR<br>**BFR**>nonBFR<br>BFR=nonBFR<br>BFR=nonBFR	+<br>=<br>=<br>=<br>+<br>+<br>+<br>+<br>=<br>=	N/A	N/A
21	Maga M. et al.	Stimulation of the vascular endothelium and angiogenesis by blood flow restricted exercise	2022	International Journal of Environmental Research and Public Health	19(23):15859	Poland	cross-over study	35	35	45,71%	24.29 ±2.44	healthy, young	interval cross-trainer cycling	1 single session	21	9-minute warm-up (10% 1RM), 6 30-seconds-long sprints (20% 1RM) with 90-second cool-downs (10% 1RM).	21	pressure cooling cuffs	Vasper TM	40mmHg (arms) and 65mmHg (legs)	same training without BFR	FMD<br>RHI<br>SI<br>RI<br>AI75<br>VEGFR-2<br>PECAM-1 (CD31)<br>CD34	**BFR>nonBFR**<br>BFR=nonBFR<br>**BFR<nonBFR**<br>**BFR<nonBFR**<br>BFR=nonBFR<br>**BFR>nonBFR**<br>**BFR>nonBFR**<br>**BFR>nonBFR**	+<br>=<br>-<br>-<br>=<br>+<br>+<br>+	N/A	N/A
22	Montgomery R. et al.	Blood Flow Restriction Exercise Attenuates the Exercise-Induced Endothelial Progenitor Cell Response in Healthy, Young Men.	2019	Frontiers in Physiology	0,727083333	UK	cross-over study	9	9	100	21 ± 1	healthy, young	resistance exercise	1 single session	N/A	1 set of 30 repeats, 3 sets of 15 reps (20% 1RM)	N/A (during whole training)	pressure cuff	Hokanson CC17 Thigh Cuff	60% of SBP, 5 mmHg in non-BFR group	same training without BFR	CD34+CD45dim<br>CD34+VEGFR2+<br>CD34+CD45dimVEGFR2+	BFR=nonBFR<br>BFR=nonBFR<br>BFR<**nonBFR**<br>BFR<**nonBFR**<br>BFR=nonBFR<br>**BFR<nonBFR**	=<br>=<br>=<br>=<br>=<br>=	N/A	N/A
23	Paiva F. M. et al.	Effects of disturbed blood flow during exercise on endothelial function: a time course analysis.	2016	Brazilian Journal of Medical and Biological Research	49:e5100	Brazil	cross-over-like study	9	9	100	28 ± 5.8	healthy, young	handgrip	1 single session	20	15 contractions / minute (60%MVC)	20	pressure cuff	E20 Rapid Cuff Inflator	80 mmHg	same training without BFR on other leg	FMD mm<br>FMD %<br>FMD%/AUC	**BFR<nonBFR**<br>N/D<br>**BFR<nonBFR**<br>BFR=nonBFR<br>**BFR<nonBFR**<br>BFR=nonBFR	=<br>=<br>=<br>=<br>-<br>-	N/A	N/A

24	Patterson S. D. et al.	Circulating hormone and cytokine response to low-load resistance training with blood flow restriction in older men.	2013	European Journal of Applied Physiology	113;:713-719	UK	cross-over study	7	7	100%	71.0 ± 6.5	healthy, older	resistance exercise	1 single session	min wysiłku + 12(	5 sets (20 % 1-RM) unknown nr of repeats	8–10	pressure cuffs	regular blood pressure cuffs	110 mmHg	same without BFR	VEGF	BFR=nonBFR<br>**BFR>nonBFR**<br>**BFR>nonBFR**	+<br>+<br>+	N/A	N/A
25	Pinto R.R. et al.	Haemodynamic responses during resistance exercise with blood flow restriction in hypertensive subjects	2015	Clinical Physiology and Functional Imaging	36(5):407-13	Brazil	cross-over study	12	12	0%	57 ± 7	women with hypertension	resistance exercise	1 single session	N/A	3 sets of 15 repeats with 30s rest between sets (20% of 1 RM)	N/A (during whole training)	pressure cuffs	regular blood pressure cuffs	100% SBP	same without BFR and high resistance without BFR	SVR	BFR= LI nonBFR<br>BFR= HI nonBFR	=	**SBP: BFR> LI nonBFR**<br>SBP: BFR= HI nonBFR<br>HR: BFR= LI nonBFR<br>HR: BFR= HI nonBFR	=<br>=
26	Ramis T. R. et al.	Effects of Traditional and Vascular Restricted Strength Training Program With Equalized Volume on Isometric	2020	Journal of Strength and Conditioning	34:689-698	Brazil	RCT	28	15	100%	23.96 ± 2.67	healthy, young	resistance exercise	3 session/week; 8 weeks	N/A	4 sets, 21 repeats for amrs + 23 for knees	N/A (during whole training + 2 minutes)	pressure cuff	N/D	- 20 mmHg; Leg: SBP +	same training with high-load resistance without BFR	FMD<br>NOx	BFR=nonBFR<br>BFR=nonBFR	+<br>+	N/A	N/A
27	Renzi C. P. et al.	Effects of leg blood flow restriction during walking on cardiovascular function	2010	Medicine and Science in Sports and Exercise	42(4):726-32	USA	cross-over study	17	17	64,70%	26 ± 1	healthy, young	walking	1 single session	14	5 sets, 2 minutes each	17	pressure cuff	regular sphygmomanometer cuffs	160mmHg	Same training	FMD<br>FMD%/AUC<br>ABI	**BFR<nonBFR**<br>**BFR<nonBFR**<br>BFR=nonBFR	-<br>-<br>=	HR: BFR=nonBFR<br>SBP: BFR=nonBFR	=<br>=
28	Shill D.D. et al.	Experimental intermittent ischemia augments exercise-induced inflammatory cytokine production	2017	Journal of Applied Physiology	123:434-441	USA	cross-over study	14	14	100%	21.8 ± 0.4	healthy, young	handgrip	1 single session	30	20 repreats / 1 minute (65% 1RM)	30	pressure cuff	Hokanson E20 cuff inflator	95% SBP	same without BFR	bFGF<br>VEGF	**BFR>nonBFR**<br>**BFR>nonBFR**<br>**BFR>nonBFR**<br>**BFR>nonBFR**	+<br>+<br>+<br>+	N/A	N/A
29	Shimizu R. et al.	Low-intensity resistance training with blood flow restriction improves vascular endothelial function and peripheral blood circulation in healthy elderly people.	2016	European Journal of Applied Physiology	116:749-757	Japan	RCT	40	20	82,50%	71 ± 4	healthy, older	resistance exercise	3 sessions / week; 4 weeks	15	3 sets of 20 repeats	15	pressure cuff	Tourniquet 9000 VBM Medizintechnik GmbH	100% SBP	same without BFR	VEGF<br>vWF<br>RHI<br>tcPO2	**BFR>nonBFR**<br>BFR=nonBFR<br>**BFR>nonBFR**<br>BFR=nonBFR	+<br>-<br>+<br>+	**HR: BFR>nonBFR**<br>**SBP: BFR>nonBFR**	+<br>+
30	Stray-Gundersen S. et al.	Walking With Leg Blood Flow Restriction: Wide-Rigid Cuffs vs. Narrow-Elastic Bands.	2020	Frontiers in Physiology	11:568	USA	cross-over study	15	15	60%	23 ± 2	healthy, young	walking	N/A	15	5 sets of 2-min walking intervals (0.9 m/s, 1-min rest between sets)	10	pressure cuff or elastic bands	Pressure cuffs: Hokanson, CC17; bands: BFR leg bands Bstrong	cuff: 160 mmHg; band: 300 mmHg	same without BFR	FMD<br>CAVI	BFR=nonBFR<br>BFR=nonBFR<br>BFR=nonBFR<br>BFR=nonBFR	=<br>=<br>=<br>=	SBP: BFR-elastic=nonBFR<br>**SBP: BFR-rigid>nonBFR**<br>HR: BFR-elastic=nonBFR<br>**HR: BFR-rigid>nonBFR**	=<br>+<br>+<br>+
31	Tai Y.L. et al.	Hemodynamic response and pulse wave analysis after upper- and lower-body resistance exercise with and	2021	European Journal of Sport Science	10:1-10	USA	cross-over study	23	23	100%	N/D	healthy young	resistance exercise	1 single session	N/A	4 sets of 30, 15, 15, and 15 repeats (30% 1RM)	N/D	pressure cuff	N/D	N/D	same high resistance	AI<br>AI75	BFR=nonBFR<br>BFR=nonBFR	-<br>-	SBP: BFR=nonBFR<br>HR: BFR=nonBFR	+<br>+
32	Takano H. et al.	Hemodynamic and hormonal responses to a short-term low-intensity resistance exercise with the reduction of	2005	European Journal of Applied Physiology	95:65-73	Japan	cross-over study	11	11	100%	34 ± 6	healthy, young	resistance exercise	1 single session	N/A	4 sets of 30 repeats	N/A	pressure cuff	KAATSU Master	160–180 mmHg	same without BFR (only 9 participants, 2 LtFU)	VEGF	**BFR>nonBFR**<br>**BFR>nonBFR**	+<br>+	**HR: BFR>nonBFR**<br>**SBP: BFR>nonBFR**	+<br>+
33	Tangchaisuriya P. et al..	Physiological Adaptations to High-Intensity Interval Training Combined with Blood Flow Restriction in Masters Road Cyclists	2022	Medicine and Science in Sports and Exercise	54(5):830-840	Thailand	RCT	50	17	100%	40.9 ± 4.3	healthy, young cyclists	cycling ergometer	6 session/week for 12 weeks	32-120	120 min cycling 55%–60%PPO (2/week) + 75 min cycling 65%–70% PPO (2/week) + 2 repeats of 4 min interval 80% PPO, 2 BFR repeats of 60% PPO with 2 min 30% PPO and 10-min cool-down 25% PPO (2/week)	N/A (during whole training + 2 minutes)	pressure cuffs	regular blood pressure cuffs	30% SBP	LI: 120 min cycling 55%–60%PPO (2/week) + 75 min cycling 65%–70% PPO (4/week)<br>or<br>HI: 120 min cycling 55%–60%PPO (2/week) + 75 min cycling 65%–70% PPO (2/week) + 4 BFR repeats of 4 min interval 80% PPO with 2 min 30% PPO and 10-min cool-down 25% PPO (2/week)	FMD<br>IMT<br>PWV<br>Shear rate<br>RHF<br>VEGF	BFR=nonBFR LI<br>BFR=nonBFR HI<br>BFR=nonBFR LI<br>BFR=nonBFR HI<br>BFR=nonBFR LI<br>BFR=nonBFR HI<br>BFR=nonBFR LI<br>BFR=nonBFR HI<br>BFR=nonBFR LI<br>BFR=nonBFR HI<br>BFR=nonBFR LI<br>BFR=nonBFR HI	=<br>=<br>=<br>=<br>=<br>+	SBP: BFR=nonBFR-LI<br>SBP: BFR=nonBFR-HI<br>HR: BFR=nonBFR-LI<br>HR: BFR=nonBFR-HI	=<br>=
34	Wooten S. V. et al.	Hemodynamic and Pressor Responses to Combination of Yoga and Blood Flow Restriction	2020	International Journal of Sports Medicine	41:759-765	USA	cross-over study	20	20	50%	23 ± 4	healthy, young	yoga	1 single session	10	20 yoga poses	10	pressure cuffs	BStrong BFR	250-300 mmHg	same without BFR	FMD<br>CAVI	BFR=nonBFR<br>BFR=nonBFR	=<br>-	HR: BFR=nonBFR<br>SBP: BFR=nonBFR	+<br>+
35	Yasuda T. et al.	Effects of detraining after blood flow-restricted low-load elastic band training on muscle size and arterial stiffness in older women.	2015	SpringerPlus	4:348	Japan	RCT	14	7	0%	69.5 ± 6.5	healthy, older	resistance exercise	2 sessions/week for 12 weeks	N/A	75 repeats	9,5	pressure cuffs	KAATSU Master	1st day - 120 mmHg +20mmHg for each next day up to 270 mmHg (mean pressure was 202 ± 8 mmHg)	same trainingwithout occlusion	FMD<br>CAVI<br>ABI	BFR=nonBFR<br>BFR=nonBFR<br>BFR=nonBFR<br>BFR=nonBFR<br>BFR=nonBFR<br>BFR=nonBFR	=<br>=<br>=<br>=<br>=<br>=	HR: BFR=nonBFR<br>HR: BFR=nonBFR<br>SBP: BFR=nonBFR<br>SBP: BFR=nonBFR	=<br>=<br>=<br>=<br>=<br>=
36	Yasuda T. et al.	Effects of Low-Load, Elastic Band Resistance Training Combined With Blood Flow Restriction on Muscle Size and Arterial Stiffness in Older Adults.	2015	The Journals of Gerontology. Series A, Biological Sciences	70:950-958	Japan	RCT	17	9	17,7%	70.01 ± 5.68	healthy, older	resistance exercise	2 sessions/week; 12 weeks	N/A	75 repeats	11	pressure cuff	KAATSU Master	180-270 mmHg	same without BFR	FMD<br>CAVI<br>ABI	BFR=nonBFR<br>BFR=nonBFR<br>BFR=nonBFR	=<br>=<br>=	HR: BFR=nonBFR<br>SBP: BFR=nonBFR	=<br>=
37	Yasuda T. et al.	Thigh muscle size and vascular function after blood flow-restricted elastic band training in older women	2016	Oncotarget	7(23):33595-607	Japan	RCT	30	20	0%	70.0 ± 6.33	healthy women	resistance exercise	2 sessions/week; 12 weeks	N/A	75 repeats	11	pressure cuff	KAATSU Master	160-200 mmHg	no training	CAVI<br>ABI<br>AI	BFR-HI=control<br>BFR-LI=control<br>BFR-HI=control<br>BFR-LI=control<br>BFR-HI=control<br>BFR-LI=control	=<br>=<br>=<br>=<br>=<br>=	HR: BFR-HI=control<br>HR: BFR-LI=control<br>cSBP: BFR-HI=control<br>cSBP: BFR-LI=control<br>bSBP: BFR-HI=control<br>bSBP: BFR-LI=control<br>aSBP: BFR-HI=control<br>aSBP: BFR-LI=control	=<br>=<br>=<br>=<br>=<br>=<br>=<br>=
38	Zhao Y. et al.	Eight weeks of resistance training with blood flow restriction improve cardiac function and vascular endothelial function in healthy young Asian males	2020	International Health	13(5):471-479	China	RCT	24	8<br>8	100%	20.63 ± 0.88	healthy, young	resistance exercise	5 sessions/week for 8 weeks	N/A	20 repeats/min/set in 5 sets, with a 2min break (30% 1RM)	N/A (during whole training)	pressure cuff	regular sphygmomanometer cuffs	65% SBP<br>130% SBP	same without BFR<br>same without BFR	VEGF-A<br>VEGFR<br>VEGF-A<br>VEGFR	**BFR-LP>nonBFR**<br>BFR-LP=nonBFR<br>BFR-HP=nonBFR<br>**BFR-HP>nonBFR**	+<br>+<br>+<br>+	SBP BFR-LP=nonBFR<br>HR BFR-LP=nonBFR<br>SBP BFR-HP=nonBFR<br>HR BFR-HP=nonBFR	=<br>-<br>=<br>-
